# Maternal Influenza Immunization and Reduced Likelihood of Prematurity and Small for Gestational Age Births: A Retrospective Cohort Study

**DOI:** 10.1371/journal.pmed.1000441

**Published:** 2011-05-31

**Authors:** Saad B. Omer, David Goodman, Mark C. Steinhoff, Roger Rochat, Keith P. Klugman, Barbara J. Stoll, Usha Ramakrishnan

**Affiliations:** 1 Hubert Department of Global Health, Emory University Rollins School of Public Health, Atlanta, Georgia, United States of America; 2 Emory Vaccine Center, Atlanta, Georgia, United States of America; 3 Georgia Department of Community Health, Atlanta, Georgia, United States of America; 4 Department of Pediatrics, Cincinnati Children's Hospital Medical Center, Cincinnati, Ohio, United States of America; 5 Department of Pediatrics, Emory University School of Medicine and Children's Healthcare of Atlanta, Atlanta, Georgia, United States of America; Mahidol University, Thailand

## Abstract

**Background:**

Infections during pregnancy have the potential to adversely impact birth outcomes. We evaluated the association between receipt of inactivated influenza vaccine during pregnancy and prematurity and small for gestational age (SGA) births.

**Methods and Findings:**

We conducted a cohort analysis of surveillance data from the Georgia (United States) Pregnancy Risk Assessment Monitoring System. Among 4,326 live births between 1 June 2004 and 30 September 2006, maternal influenza vaccine information was available for 4,168 (96.3%). The primary intervention evaluated in this study was receipt of influenza vaccine during any trimester of pregnancy. The main outcome measures were prematurity (gestational age at birth <37 wk) and SGA (birth weight <10th percentile for gestational age). Infants who were born during the putative influenza season (1 October–31 May) and whose mothers were vaccinated against influenza during pregnancy were less likely to be premature compared to infants of unvaccinated mothers born in the same period (adjusted odds ratio [OR] = 0.60; 95% CI, 0.38–0.94). The magnitude of association between maternal influenza vaccine receipt and reduced likelihood of prematurity increased during the period of at least local influenza activity (adjusted OR = 0.44; 95% CI, 0.26–0.73) and was greatest during the widespread influenza activity period (adjusted OR = 0.28; 95% CI, 0.11–0.74). Compared with newborns of unvaccinated women, newborns of vaccinated mothers had 69% lower odds of being SGA (adjusted OR = 0.31; 95% CI, 0.13–0.75) during the period of widespread influenza activity. The adjusted and unadjusted ORs were not significant for the pre-influenza activity period.

**Conclusions:**

This study demonstrates an association between immunization with the inactivated influenza vaccine during pregnancy and reduced likelihood of prematurity during local, regional, and widespread influenza activity periods. However, no associations were found for the pre-influenza activity period. Moreover, during the period of widespread influenza activity there was an association between maternal receipt of influenza vaccine and reduced likelihood of SGA birth.

*Please see later in the article for the Editors' Summary*

## Abstract 2

In an analysis of surveillance data from the state of Georgia (US), Saad Omer and colleagues show an association between receipt of influenza vaccination among pregnant women and reduced risk of premature births.

## Editors' Summary

**Background:**

Maternal infections during pregnancy can have harmful effects on both mother and baby. For example, influenza is associated with increased morbidity and mortality among pregnant women compared to women who are not pregnant or who acquire influenza infection after delivery. And some respiratory infections, especially those that can cause maternal pneumonia such as influenza virus, are known to be associated with the baby being small—below the 10th percentile—for gestational age and with an increased risk of preterm birth—birth before 37 weeks of gestation. Previous studies have shown that inactivated influenza vaccination during pregnancy provides protection against influenza virus for both mother and baby. As there has been an increase in the rate of preterm birth the United States from 9.5% in 1981 to 12.8% in 2006, the impact of maternal influenza immunization on birth outcomes has important public health implications and is of particular interest during influenza pandemics.

**Why Was This Study Done?:**

Given that maternal vaccination can protect babies from influenza virus, it is plausible that influenza vaccination in pregnancy could mitigate adverse birth outcomes such as prematurity and the baby being small for gestational age. The researchers of this study set out to evaluate this hypothesis by investigating whether there was an association between women receiving inactivated influenza vaccine during pregnancy and positive birth outcomes for their babies in the population of the state of Georgia, in the United States.

**What Did the Researchers Do and Find?:**

The researchers conducted a retrospective cohort analysis of a large surveillance dataset (the Georgia Pregnancy Risk Assessment Monitoring System) to analyze the relationship between receipt of inactivated influenza vaccine during any trimester of pregnancy by mothers of infants born between June 1, 2004, and September 30, 2006, and their baby being premature or small for gestational age. The study period encompassed the 2004–2005 and 2005–2006 influenza seasons—the two most recent seasons for which the data were available. The researchers did a stratified analysis for the overall study period, and various periods during it, and also weighted their analysis to adjust for possible oversampling. They used logistic regression to evaluate the association of maternal influenza vaccine and (a) prematurity and (b) small for gestational age, and also used linear regression to evaluate the statistical significance of differences between vaccinated and unvaccinated women for mean gestational age at first antenatal visit and mean birth weight.

During the study period, 4,168 mother–baby pairs were included in the analysis. Local influenza activity was detected during 27 weeks (22.1%), and 578 women (14.9% [weighted]) had received the influenza vaccine during pregnancy, giving a vaccination coverage of 19.2% (weighted) among mothers of infants born during the assumed influenza season. In the study sample, 1,547 babies (10.6% [weighted]) were born premature, and 1,186 babies (11.2% [weighted]) were small for gestational age. Infants who were born during the assumed influenza season (October–May) and whose mothers were vaccinated against influenza during pregnancy were less likely to be premature than infants of unvaccinated mothers born in the same period, with an adjusted odds ratio of 0.60. The effect of maternal influenza vaccine on reducing prematurity was the highest for infants born during the period of widespread influenza activity, with 72% lower odds of prematurity in infants of vaccinated mothers than infants of unvaccinated mothers. Compared with newborns of unvaccinated women, babies of vaccinated mothers also had 69% lower odds of being small for gestational age during the period of widespread influenza activity, but the adjusted and unadjusted odd ratios were not significant for the pre-influenza activity period.

**What Do These Findings Mean?:**

These results show that there was an association between maternal immunization with the inactivated influenza vaccine during pregnancy and reduced likelihood of prematurity during local, regional, and widespread influenza activity periods. In addition, during the period of widespread influenza activity there was an negative association between maternal receipt of influenza vaccine and small for gestational age birth.

**Additional Information:**

Please access these Web sites via the online version of this summary at http://dx.doi.org/10.1371/journal.pmed.1000441.

- More information about influenza vaccination during pregnancy is available from the World Health Organization and the UK National Health Service
- More information about the Georgia Pregnancy Risk Assessment Monitoring System is also available

## Introduction

Infections during pregnancy have the potential to adversely impact birth outcomes and fetal growth and development. Respiratory infections—particularly those resulting in pneumonia—have been associated with low birth weight and increased risk of preterm birth [1],[2]. Influenza virus is an important respiratory pathogen that causes substantial burden of disease—including morbidity and mortality among pregnant women, with greater risk of influenza-related morbidity among pregnant women than among non-pregnant and postpartum women [3].

Vaccination against influenza is the most effective tool to prevent morbidity and mortality due to influenza. Influenza vaccination during pregnancy provides protection for the infant as well as the mother. A randomized controlled clinical trial in Bangladesh demonstrated that vaccination of pregnant women with the inactivated influenza vaccine had 63% effectiveness in reducing laboratory-confirmed influenza in their infants [4]. Since there is evidence of adverse fetal/newborn outcomes after infection during pregnancy [5],[6], including influenza infection [2], it is plausible that influenza vaccination in pregnancy could mitigate adverse birth outcomes such as prematurity and small for gestational age (SGA) births. The potential impact of maternal influenza immunization on birth outcomes could have important public health implications for developed as well as developing countries and may be of particular interest during influenza pandemics.

The objective of this study was to evaluate whether there is an association between receipt of inactivated influenza vaccine during pregnancy and birth outcomes using a multiyear, population-based, state-wide dataset from the state of Georgia (in the United States).

## Methods

We conducted a retrospective cohort analysis of a large surveillance dataset. The primary exposure variable was receipt of inactivated influenza vaccine during any trimester of pregnancy by mothers of infants born between 1 June 2004 and 30 September 2006. The study period encompassed the 2004–2005 and the 2005–2006 influenza seasons (the two most recent seasons for which the data were available at the time of analysis). The main outcomes assessed were prematurity and SGA.

### Data Sources and Study Population

We analyzed pregnancy- and birth-related data from the Georgia Pregnancy Risk Assessment Monitoring System (PRAMS) and influenza activity information compiled by Georgia for the Council of State and Territorial Epidemiologists (CSTE) reports. PRAMS is a multistate surveillance system managed by the US Centers for Disease Control and Prevention and state health departments, including the Georgia Department of Community Health [7],[8]. The PRAMS sample is drawn monthly from the state's birth file and includes resident women who have experienced a live birth. Georgia PRAMS oversamples women based on race (black) and birth weight (<2,500 g). We adjusted for the oversampling by using analysis weights described elsewhere in the methods section. PRAMS data are collected primarily by mailed questionnaires, with telephone follow-up among non-responders.

The Georgia PRAMS dataset contains information on maternal influenza vaccination (during any trimester); maternal attitudes, behaviors, and experiences before, during, and shortly after pregnancy; and newborn birth certificate data, including birth date and birth weight. Prematurity was defined as clinical estimate of gestational age (at birth) as less than 37 wk. Newborns with birth weight below the10^th^ percentile for their gestational age were considered to be SGA. We used gender-specific reference values for the US published by Oken at al. [9] to assign SGA (yes/no) categories.

### Definitions of Influenza Activity Periods

**Figure 1:**
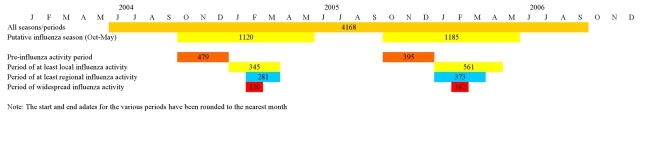
Influenza activity/analysis periods. The numbers indicate births in each of the analysis periods.

In order to model the impact of the intensity of influenza activity in Georgia on the association between maternal influenza vaccination and birth outcomes, we used a modified version of the CSTE report categories of influenza activity. CSTE reports assess the spread of influenza within each state for each week based on lab-confirmed and syndromic data [10]. The influenza activity is considered local if there are influenza outbreaks or an increase in cases of influenza-like illness in a single region of the state along with recent identification of laboratory-confirmed influenza from that region. In the case of influenza outbreaks or increases in influenza-like illness with recent laboratory-confirmed influenza in at least two but fewer than half the regions of the state, the influenza activity level is considered to be regional. If the influenza outbreaks or increased numbers of influenza-like illness cases (plus laboratory-confirmed cases) are reported in at least half of the regions of the state, the influenza activity is considered to be widespread (Figure 1). We defined the pre-influenza period as the period between the start of the putative influenza season (October 1) and the beginning of local influenza activity (per CSTE reports). The pre-influenza period is characterized by the availability of the vaccine and the absence of influenza activity (Figure 1). The definition for the pre-influenza period was similar to the one used by other authors [11],[12].

Stratified analysis was performed for the overall study period, the putative influenza season (1 October–31 May), the pre-influenza period (during the putative influenza season), the period of at least local activity, the period of regional activity, and the period of widespread activity (Figure 1).

### Statistical Analysis and Confounder Assessment

In Georgia PRAMS, live births to black women and those that are low birth weight are oversampled in order to provide enough statistical power for stratified analyses for groups of interest relevant to PRAMS objectives. For combined analyses, in accordance with standard practice, analysis weights developed by the Centers for Disease Control and Prevention [13] were used to adjust for the sample design (e.g., to account for oversampling of the two risk groups) and differential response rate across groups.

We used logistic regression to evaluate the association of maternal influenza vaccine and (a) prematurity and (b) SGA. Linear regression was used to evaluate the statistical significance of differences between infants born to vaccinated and unvaccinated women in terms of mean gestational age at first antenatal visit and mean birth weight.

Confounding due to differences between the vaccinated and unvaccinated individuals is a recognized issue in observational studies of influenza vaccination—particularly studies evaluating vaccine effectiveness for reducing all-cause mortality in the elderly [12]. One approach to account for confounding is to choose a period when the vaccine was available but the influenza virus was not circulating as the “control” pre-influenza period [11],[12]. In the pre-influenza period, there should be no vaccine effect, and observed effects during this period are assumed to be due to confounding. Therefore, as our primary strategy for confounder adjustment, we identified a group of covariates that would move the odds ratios (ORs) of association between maternal influenza immunization and birth outcomes during the pre-influenza period to 1.0 (i.e., no effect), hence arriving at a set of covariates that could effectively control for confounding due to the differences between the vaccinated and the unvaccinated women in analyses of all influenza activity periods. We selected covariates for the separate multivariate models for each of the birth outcomes (i.e., prematurity and SGA) using a modified version of the approach described by Jackson et al. [11] and Nelson et al. [12]. Briefly, variables for each birth outcome were evaluated for potential confounding by selecting the covariates that, in bivariate models, modified the association between maternal influenza vaccine and the birth outcome and moved the OR towards 1. From this initial group of variables, we arrived at a parsimonious model by sequentially dropping each covariate and observing a change in OR of the association between maternal immunization and the birth outcome. We excluded the variable whose removal moved the OR the most towards 1. If dropping a covariate resulted in moving ORs away from 1 and a change in magnitude of less than 1%, we removed the covariate that caused the least change and then repeated the process. If the change was more than 1%, we considered the current set of covariates as the smallest group of variables required to account for confounding.

In order to address the possibility that the confounders in the pre-influenza period were different from the confounding factors in the influenza activity period, we also developed secondary multivariate models using a traditional analytic approach for confounder adjustment. In these secondary models, the covariate list (for both the pre-influenza-period-based and traditional adjustment) was based on evidence in the literature regarding associations with birth outcomes and availability of data in the Georgia PRAMS dataset. The covariate list included the following: gestational age at first antenatal visit, maternal age less than 19 y, maternal age more than 35 y, multiple births, maternal medical risk factors, labor/delivery complications, birth defects, maternal diabetes (gestational and/or non-gestational), hypertension (treated or untreated), mother insured, multivitamin use in pregnancy, history of smoking during pregnancy, history of alcohol use during pregnancy, black race, education less than 12^th^ grade, mother's marital status, and maternal weight before pregnancy. The ORs for association between the different covariates and receipt of influenza vaccine were computed using logistic regression.

We also computed the population prevented fraction of prematurity for the various periods of influenza activity using the formula: 
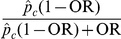
 where 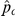 is the proportion of cases vaccinated and OR is the odds ratio approximating relative risk (we verified this assumption for our data). The formula, based on the approach described by Kleinbaum et al. [14], is suitable for computing the population prevented fraction when adjusted measures of association are used. The population prevented fraction for a vaccine estimates the reduction in an outcome given the efficacy/effectiveness of the vaccine and the specific vaccine coverage.

We used Stata version 10 (Stata Corporation) for statistical analysis. Identified associations were evaluated for statistical significance at α = 0.05 using two-tailed tests, and Taylor series linearization methods [15] were used to estimate variance.

### Ethical Assessment and Institutional Review Boards

The study was reviewed and approved by the Emory University Institutional Review Board and the Georgia Department of Human Resources Institutional Review Board.

## Results

A total of 4,326 women (and their newborns) were included in Georgia PRAMS during the 28-mo study period. Influenza vaccine information was available for 4,168 (96.3%) women in PRAMS (study population); of these, 578 women (14.9% [weighted]) had received the influenza vaccine during pregnancy. The vaccine coverage was 19.2% (weighted) among mothers of infants born during the putative influenza season. Out of the 122 wk of the study, at least local influenza activity was detected during 27 wk (22.1%)—including widespread activity in 8 wk (Figure 1). There were 1,547 premature newborns (10.6% [weighted]) and 1,186 newborns with SGA (11.2% [weighted]) in our study population.

The odds of having received influenza vaccine during pregnancy were lower (a) for black women than for all other ethnic groups (OR = 0.78; 95% CI, 0.62–0.98), (b) for women with diabetes (OR = 0.30, 95% CI, 0.10–0.95), and (c) for mothers who used multivitamins in pregnancy (OR = 0.64; 95% CI, 0.50–0.83) (Table 1). Insured women were more likely to have received the influenza vaccine (OR = 1.41; 95% CI, 1.09–1.81). Likelihood of having received an influenza vaccine during pregnancy was not associated with any of the other binary covariates (Table 1). Similarly, gestational age at first prenatal visit was similar for vaccinated women and unvaccinated women (mean: 5.2 wk versus 5.3 wk; *p* = 0.23), and maternal weight before pregnancy was similar for vaccine recipients and non-recipients (mean: 68.3 kg versus 68.1 kg; *p* = 0.88).

**Table 1:** Receipt of influenza vaccine during pregnancy categorized by maternal characteristics.

Characteristic	Maternal/Demographic Category: Yes		Maternal/Demographic Category: No		*p*-Value	OR (95% CI)^a^
	Vaccinated^b^ [*n* (%)]	Total^c^ [*n* (%)]	Vaccinated^b^ [*n* (%)]	Total^c^ [*n* (%)]		
Maternal age less than 19 y	92 (18.3)	536 (11.5)	486 (14.5)	3,632 (88.5)	0.13	1.32 (0.92–1.89)
Maternal age more than 35 y	79 (17.7)	521 (12.5)	499 (14.6)	3,647 (87.5)	0.2	1.26 (0.89–1.79)
Multiple births (during current gestation)	184 (9.0)	282 (1.9)	393 (15.1)	3,876 (98.1)	0.11	0.56 (0.27–1.16)
Maternal medical risk factors^d^	184 (13.8)	1,486 (29.6)	393 (15.5)	2,689 (70.4)	0.34	0.88 (0.66–1.15)
Labor/delivery complications^e^	248 (16.3)	1,735 (32.4)	330 (14.3)	2,433 (67.6)	0.23	1.17 (0.91–1.51)
Birth defect	16 (15.4)	87 (1.4)	562 (15.0)	4,081 (98.6)	0.94	1.03 (0.41–2.61)
Maternal diabetes	10 (5.2)	100 (2.0)	567 (15.2)	4,057 (98.0)	0.03	0.30 (0.08–0.95)
Hypertension	39 (15.1)	316 (4.1)	538 (15.0)	3,841 (95.9)	0.97	1.01 (0.57–1.78)
Mother insured	325 (17.1)	2,099 (52.1)	253 (12.7)	2,060 (47.9)	0.0007	1.41 (1.09–1.81)
Multivitamin use in pregnancy	324 (12.7)	2,587 (60.1)	252 (18.4)	1,565 (39.4)	0.0005	0.64 (0.50–0.83)
History of smoking during pregnancy	31 (9.3)	306 (6.9)	545 (15.4)	3,847 (93.1)	0.06	0.56 (0.31–1.02)
History of alcohol use during pregnancy	7 (31.9)	33 (0.7)	569 (14.8)	4,120 (99.30)	0.08	2.7 (0.86–8.43)
Black race	282 (12.9)	2,117 (31.9)	294 (15.9)	2,035 (68.1)	0.03	0.78 (0.62–0.98)
Education less than 12th grade	95 (16.1)	610 (17.3)	420 (13.7)	3,226 (82.7)	0.28	1.21 (0.85–1.71)
Mother married	307 (15.6)	2,219 (58.5)	271 (14.1)	1,945 (41.5)	0.36	1.22 (0.87–1.44)

All percentages calculated with analytical weights.

a Ratio of the odds of having received influenza vaccine by mothers in each binary (yes/no) category of a covariate, e.g., the odds of having received an influenza vaccine were 22% higher among married women than among unmarried women.

b Number and proportion of mothers in each of the binary (yes/no) category of a covariate who received the influenza vaccine, e.g., 14.1% of unmarried mothers had received the influenza vaccine compared to 15.6% of married women.

c Total (and weighted percent) of respondants with “Yes” or “No” in a maternal/demographic characteristic category (out of all respondents).

d Medical risk factors include acute or chronic lung disease; anemia (hemoglobin <10 gm/dl or hematocrit <30%); cardiac disease; diabetes; eclampsia; genital herpes; hemoglobinopathy; hydramnios/oligohydramnios; chronic hypertension; pregnancy-induced hypertension, incompetent cervix; previous infant >4,000 g; previous preterm, SGA, or low birth weight delivery; renal disease; Rh sensitization; rubella; syphilis; and uterine bleeding.

e Labor/delivery complications include abruptio placenta, anesthetic complications, breech presentation, cephalopelvic disproportion, cord prolapse, dysfunctional labor, excessive bleeding, febrile (100°F/38°C), fetal distress, moderate to heavy meconium staining, placenta previa, labor <3 h, premature rupture of membranes >12 h, labor >20 h, and seizures during labor.

Based on the approach of identifying covariates that produce adjusted ORs of 1 during the pre-influenza period, the group of covariates in the prematurity multivariate models included gestational age for first antenatal visit, maternal diabetes (gestational and/or non-gestational), multivitamin use in pregnancy, history of alcohol use during pregnancy, education less than 12th grade, and mother married. The covariates in the primary multivariate models for SGA included maternal age less than 19 y, maternal medical risk factors, labor/delivery complications, hypertension (treated or untreated), birth defects, and history of alcohol use during pregnancy.

In unadjusted models, and in models with covariates based on lack of effects in the pre-influenza season, infants born during the putative influenza season (1 October–31 May) and whose mothers were vaccinated against influenza during pregnancy were less likely to be premature than infants of unvaccinated mothers born in the same period (adjusted OR = 0.60; 95% CI, 0.38–0.94). The magnitude of effect of maternal influenza vaccine on prematurity increased during the period when there was at least local influenza activity in any part of the state (adjusted OR = 0.44; 95% CI, 0.26–0.73) and was the highest for those born during the period of widespread influenza activity (adjusted OR = 0.28; 95% CI, 0.11–0.74) (Table 2). The adjusted and unadjusted ORs were not significant for the association between receipt of maternal influenza vaccine and prematurity for the pre-influenza activity period or for the analysis without consideration of influenza activity (Table 2).

**Table 2:** ORs of prematurity by maternal influenza vaccine status (ORs<1 imply a protective association of the vaccine).

Analysis Period	Unadjusted Models		Primary Adjusted Models^a^		Secondary Adjusted Models^b^	
	OR (95% CI)^c^	*p*-Value	OR (95% CI)^c^	*p*-Value	OR (95% CI)^c^	*p*-Value
All seasons/periods	0.75 (0.54–1.04)	0.09	0.82 (0.57–1.18)	0.28	0.83 (0.55–1.26)	0.38
Putative influenza season (Oct–May)	0.60 (0.41–0.89)	0.01	0.60 (0.38–0.94)	0.02	0.54 (0.32–0.90)	0.02
Pre-influenza activity period	0.74 (0.34–1.59)	0.44	1.00 (0.39–2.54)	1.00	0.81 (0.30–2.16)	0.67
Period of at least local influenza activity	0.56 (0.33–0.96)	0.04	0.44 (0.26–0.73)	0.001	0.40 (0.24–0.68)	0.001
Period of at least regional influenza activity	0.57 (0.30–1.10)	0.10	0.41 (0.23–0.73)	0.003	0.36 (0.19–0.68)	0.002
Period of widespread influenza activity	0.34 (0.16–0.73)	0.006	0.28 (0.11–0.74)	0.01	0.27 (0.08–0.86)	0.03

a The primary adjusted models were based on the approach of identifying covariates that produce adjusted ORs of 1 during the pre-influenza period and included the following covariates: gestational age for first antenatal visit, maternal diabetes (gestational and/or non-gestational), multivitamin use in pregnancy, history of alcohol use during pregnancy, education less than 12th grade, and mother married.

b In the secondary adjusted models, the covariates included gestational age at first antenatal visit, maternal age less than 19 y, maternal age more than 35 y, multiple births, maternal medical risk factors, labor/delivery complications, birth defects, maternal diabetes (gestational and/or non-gestational), hypertension (treated or untreated), mother insured, multivitamin use in pregnancy, history of smoking during pregnancy, history of alcohol use during pregnancy, black race, education less than 12th grade, mother's marital status, and maternal weight before pregnancy. The ORs for association between the different covariates and receipt of influenza vaccine were computed using logistic regression.

c Ratio of the odds of prematurity in newborns of mothers who received influenza vaccine during pregnancy compared to mothers who did not receive the vaccine by intensity of influenza activity, e.g., in the analysis of all seasons/periods, the (unadjusted) odds of prematurity were 25% lower among the infants of mothers who received the influenza vaccine during pregnancy than among infants whose mothers who did not receive the vaccine.

Compared with newborns of unvaccinated women, those born to vaccinated mothers had lower odds of SGA (adjusted OR = 0.31; 95% CI, 0.13–0.75) during the period of widespread influenza activity (Table 3). Although the magnitude of the ORs of the association between maternal influenza vaccine and SGA generally increased with the increase in the intensity of influenza activity, these associations were not statistically significant (other than for the period of widespread activity) (Table 3). The associations observed in the secondary multivariate models using the traditional approach were qualitatively similar to the associations in the primary multivariate models (Tables 2 and 3).

**Table 3:** ORs of being SGA by maternal influenza vaccine status (ORs<1 imply a protective association of the vaccine).

Analysis Period	Unadjusted Models		Primary Adjusted Models^a^		Secondary Adjusted Models^b^	
	OR (95% CI)^c^	*p*-Value	OR (95% CI)^c^	*p*-Value	OR (95% CI)^c^	*p*-Value
All seasons/periods	0.84 (0.60–1.19)	0.33	0.83 (0.59–1.17)	0.29	0.96 (0.66–1.42)	0.86
Putative influenza season (Oct–May)	0.74 (0.47–1.15)	0.18	0.74 (0.47–1.15)	0.18	0.84 (0.50–1.40)	0.50
Pre-influenza activity period	1.07 (0.47–2.42)	0.87	1.02 (0.45–2.29)	0.96	1.02 (0.42–2.48)	0.96
Period of at least local influenza activity	0.73 (0.40–1.33)	0.30	0.74 (0.39–1.39)	0.35	0.68 (0.32–1.46)	0.33
Period of at least regional influenza activity	0.80 (0.39–1.64)	0.54	0.76 (0.34–1.71)	0.51	0.70 (0.27–1.87)	0.49
Period of widespread influenza activity	0.32 (0.14–0.73)	0.007	0.31 (0.13–0.75)	0.009	0.29 ( 0.09–0.91)	0.04

a The primary adjusted models were based on the approach of identifying covariates that produce adjusted ORs of 1 during the pre-influenza period and included the following covariates: gestational age for first antenatal visit, maternal diabetes (gestational and/or non-gestational), multivitamin use in pregnancy, history of alcohol use during pregnancy, education less than 12th grade, and mother married.

b In the secondary adjusted models, the covariates included gestational age at first antenatal visit, maternal age less than 19 y, maternal age more than 35 y, multiple births, maternal medical risk factors, labor/delivery complications, birth defects, maternal diabetes (gestational and/or non-gestational), hypertension (treated or untreated), mother insured, multivitamin use in pregnancy, history of smoking during pregnancy, history of alcohol use during pregnancy, black race, education less than 12th grade, mother's marital status, and maternal weight before pregnancy. The ORs for association between the different covariates and receipt of influenza vaccine were computed using logistic regression.

c Ratio of the odds of being SGA among newborns of mothers who received influenza vaccine during pregnancy compared to those born to mothers who did not receive the vaccine by intensity of influenza activity, e.g., in the analysis of all seasons/periods, the (unadjusted) odds of being SGA were 84% lower among the newborns of mothers who received influenza vaccine during pregnancy than among infants whose mothers who did not receive the vaccine.

Newborns of vaccinated women were, on average, 96.7 g heavier than newborns of unvaccinated women (3,348 g versus 3,251 g; *p* = 0.002). During the putative influenza season, the difference between the two groups increased to 113 g (3,360 g for the vaccinated group versus 3,247 g for the unvaccinated group; *p* = 0.004). There were no significant differences in birth weights outside the putative influenza season (3,317 g [vaccinated] versus 3,255 g [unvaccinated]; *p* = 0.233).

There was no statistical interaction by specific influenza season (i.e., 2004–2005 and 2005–2006) for all analyses of prematurity, SGA, and birth weight (for all interaction terms: *p*>0.05—detailed data available on request). Moreover, the association between maternal influenza vaccine and birth outcomes was qualitatively similar for the two influenza seasons. For example, during the period of widespread influenza activity, the adjusted ORs for prematurity were 0.17 (95% CI, 0.03–0.86) for the 2004–2005 season and 0.32 (95% CI, 0.10–1.14) for the 2005–2006 season.

The fraction of prematurity prevented in the population during the study period (population prevented fraction of prematurity) was 0% for the pre-influenza activity period and 7.9% for the putative influenza season. The population prevented fraction increased during the periods of influenza activity (at least local activity, 15.7%; at least regional activity, 17.5%; widespread activity, 17.5%).

## Discussion

This study demonstrates an association between immunization with the inactivated influenza vaccine during pregnancy and reduced likelihood of prematurity during local, regional, and widespread influenza activity periods. For births during the 8 wk of widespread influenza activity, the odds of prematurity were approximately 70% lower among the newborns of the vaccinated mothers compared to mothers who did not receive the influenza vaccine. During the period of widespread influenza activity there was also an association between maternal receipt of influenza vaccine and reduced likelihood of SGA. The magnitude of association between influenza vaccine and prematurity (as measured by the values of ORs) increased with the increase in the intensity of influenza activity and was higher for the 2004–2005 season than for the 2005–2006 season. Based on laboratory and epidemiologic criteria, the 2004–2005 influenza season was more intense than the 2005–2006 season in the US [16]. Although the SGA-related ORs were not statistically significant for influenza activity periods except for the period of widespread activity, the overall “gradient” of effect in the point estimates of the ORs was qualitatively similar to that of prematurity. The increase in the impact of maternal influenza vaccines on birth outcomes by influenza activity, both in terms of ORs and population prevented fractions, supports the validity of our findings.

The possibility of confounding due to differences between vaccinated and unvaccinated individuals included in observational studies of influenza immunization is well recognized [12]. The most significant type of confounding in influenza studies is due to a higher likelihood of individuals with high functional capacity (i.e., healthier individuals) to get vaccinated—a phenomenon often known as the “healthy user effect.” However, most observational studies where significant confounding has been documented were conducted in the elderly and included a relatively nonspecific end point of all-cause mortality. It is reasonable to assume that, compared to older individuals, women of reproductive age may be less likely to have significant functional limitation even in the presence of co-morbidities. Therefore, influenza vaccine studies in this age group may be less likely to suffer from bias due to the healthy user effect. Moreover, we found no statistically significant difference between the vaccinated women and the unvaccinated women in terms of gestational age at which they sought antenatal care. On the other hand, the possibility of other confounders cannot be discounted in studies involving pregnant women. In order to address confounding, we used the pre-influenza period (i.e., the season where vaccine was available but there was minimal circulation of influenza virus) as the “control” period. The use of the pre-influenza period for selecting confounders from a broad set of covariates is an approach suggested by Nelson et al. [12] and Jackson et al. [11] that takes advantage of the seasonality of influenza circulation. The associations observed in our study were robust to adjustment for confounders identified using this approach (and the more traditional approach)—supporting the validity of our findings.

Influenza virus, particularly seasonal influenza virus, rarely crosses the placenta [3],[17],[18]. However, the effect of infection on prematurity is thought be at least partially mediated through inflammatory pathways [5],[6]. Increase in pro-inflammatory cytokines (e.g., IL-1, IL-6 and TNF-α) and reduction in anti-inflammatory cytokines (e.g., IL-10) have been linked to preterm labor [6],[19],[20]. IL-1 stimulates the amnion and the decidua to produce prostaglandins and can stimulate myometrial contractions [20]. Prostaglandins are known to play an important role in the initiation and progression of labor [21]. Moreover, in animal models, administration of IL-1 results in preterm birth [20]. Similarly, TNF-α induces the amnion, the decidua, and the myometrium to produce prostaglandins, and administration of TNF-α to pregnant animals can induce preterm labor [19],[22]. Recent studies have shown that influenza virus infection induces gene expression of pro-inflammatory cytokines including IL-1β, IL-6, TNF-α, interferon (IFN)-β, IFN-α, and granulocyte macrophage colony-stimulating factor (GM-CSF) [19].

In addition to biological plausibility, there is epidemiological evidence of an association between maternal infection and preterm birth [5]. The association is strongest for intrauterine viral infections and systemic and intrauterine bacterial infections [5],[6]. Viral infection outside the reproductive tract, including influenza infection, may also play a role in the etiology of prematurity. For example, in an analysis of 1957–1958 data, newborns of women who had serological evidence of “Asian” (pandemic) influenza during pregnancy were 50% more likely to be premature compared to newborns of uninfected women [2]. Moreover, a recent literature review found that SARS infection in the second or third trimester of pregnancy may be associated with spontaneous preterm delivery and early cesarean sections for deteriorating medical condition, although only 16 such cases were identified in the literature [23]. Moreover, in studies in China and Hungary, birth defects were associated with history of influenza [24],[25].

However, a few observational studies have failed to demonstrate an association between influenza infection and birth outcomes [26],[27]. The lack of observed effect in some studies could be due to a true lack of association, small or difficult to measure effect size, challenges related to the study population (e.g., administrative datasets), or non-differential misclassification due to challenges in retrospectively identifying influenza infection. Although less than ideal, modeling receipt of influenza vaccine as the exposure/independent variable reduces the likelihood and the intensity of non-differential misclassification bias.

Preterm births represent a significant burden to health care and society [5]. Like several developed countries, there has been an increase in the rate of preterm births in the US, which rose from 9.5% in 1981 to 12.8% in 2006 [28],[29]. Although the etiology of prematurity is complex [5] and not completely understood, our results suggest that at least a fraction of preterm births may be preventable through maternal influenza vaccination.

The association between maternal influenza vaccination and SGA was only statistically significant (and the highest in magnitude) for the period of widespread influenza activity. Possible reasons for the effect being limited to the period of highest influenza activity include the following: (a) in a developed country setting, the effect of maternal influenza infection on fetal growth is milder than the effect on prematurity; (b) SGA represents fetal compromise resulting from infection that is insufficient to trigger early parturition, but may result in the delayed observation of growth restriction (i.e., the observation in the widespread activity period may be the cumulative effect of previous periods). Moreover, in the vaccinated group, the birth weight distribution in the pre-influenza period was different from the distribution in the period of widespread activity (see Text S1). However, the difference in mean birth weights (in the vaccinated group) between these two periods was not statistically significant (*p* = 0.74). Since the ostensible increase in birth weight in the widespread activity period compared to the pre-influenza period in the vaccinated group cannot easily be explained by vaccine action, this difference—although non-significant—may suggest confounding vis-à-vis the birth weight outcome.

This study has a few limitations and strengths. Although we assessed and adjusted for many covariates, like any observational study, there is a possibility of residual confounding and selection bias. Moreover, data on influenza infection during pregnancy were not included in the PRAMS dataset. Although the primary explanation of the effects of influenza immunization in pregnancy on birth outcomes is through prevention of infection, having influenza infection data would have provided additional support for our findings. Another issue is that the information regarding maternal influenza immunization was based on recall and could be susceptible to information bias. However, the vaccination rates in our study are similar to the rates computed by other authors for Georgia, and to the United States national level coverage estimated by the National Health Interview Survey [30],[31].

The PRAMS dataset does not contain information regarding the precise trimester of vaccination. Therefore, the effect of vaccination in a specific trimester could not be evaluated. Moreover, it is possible that mothers of premature infants had less time to receive influenza vaccine than mothers of term infants (i.e., reverse causality). On the other hand, since this was a population-based study with a sampling strategy aimed at producing representative estimates, the temporal distribution of influenza vaccination in pregnancy would be similar to that of the general population, hence adding to the generalizability of our findings. The results of this study, nevertheless, need to be replicated in other populations as it is plausible that the impact of vaccines on birth outcomes would vary with the underlying influenza epidemiology and demographic characteristics.

## Supporting Information

Text S1 Impact of maternal influenza immunization on likelihood of prematurity and SGA births.
(0.21 MB DOC)

## Abbreviations

CSTE: Council of State and Territorial Epidemiologists
OR: odds ratio
PRAMS: Pregnancy Risk Assessment Monitoring System
SGA: small for gestational age
