# Supplementary material for: Maternal Influenza Immunization and Reduced Likelihood of Prematurity and Small for Gestational Age Births: A Retrospective Cohort Study
Source: PLoS Med. 2011 May 31;8(5):e1000441. doi: 10.1371/journal.pmed.1000441 (PMC3104979; doi:10.1371/journal.pmed.1000441)
Supplement: Text S1 — Impact of maternal influenza immunization on likelihood of prematurity and SGA births. (0.21 MB DOC) [file pmed.1000441.s001.doc]

Supplementary Material

**Impact of Maternal Influenza Immunization on Likelihood of Prematurity and Small for Gestational Age Births**


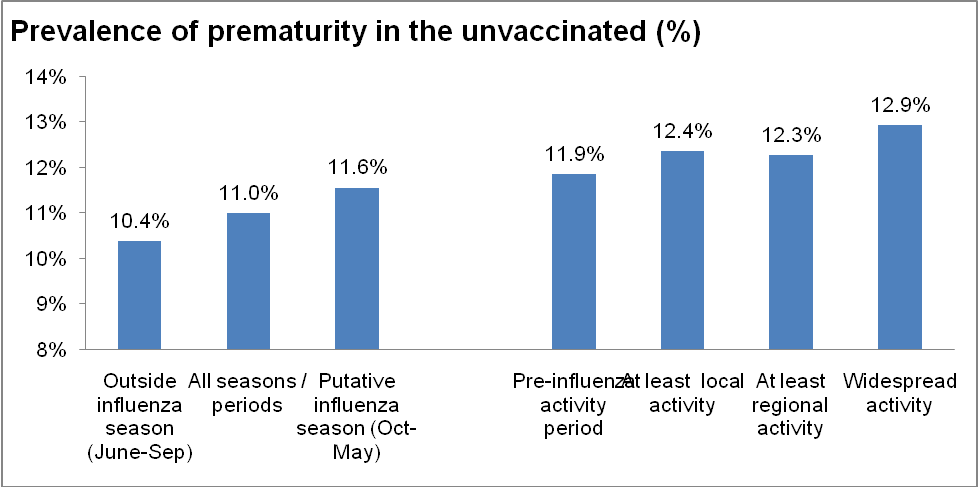


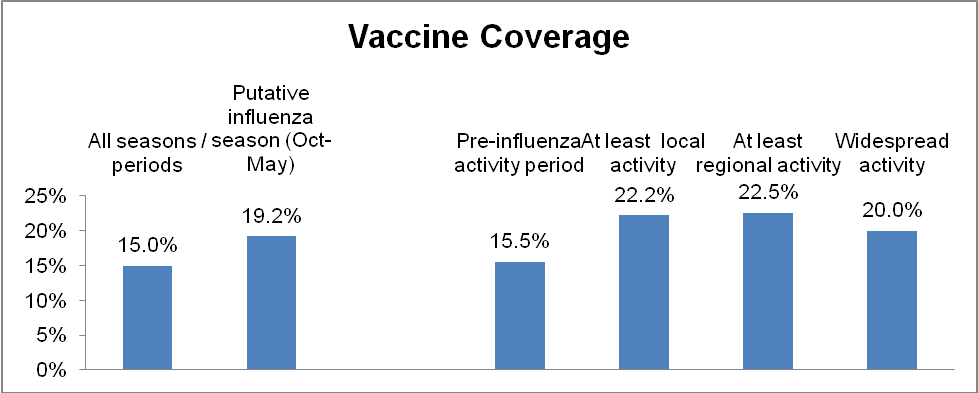


Birth Weight Percentile -Weighted

|  |  |  |
| --- | --- | --- |
|  |  |  |

| ` | | | | | | | | | | | | | |
| --- | --- | --- | --- | --- | --- | --- | --- | --- | --- | --- | --- | --- | --- |
| **Table S1: Complete results of secondary adjusted models for prematurity** | | | | | | | | | | | | | |
| Covariate | All seasons/periods | | Putative influenza season (Oct-May) | | Pre-influenza activity period | | Period of at least local influenza activity | | Period of at least regional influenza activity | | Period of widespread influenza activity | | |
|  | OR (95% CI) | p value | OR (95% CI) | p value | OR (95% CI) | p value | OR (95% CI) | p value | OR (95% CI) | p value | OR (95% CI) | | p value |
|  |  |  |  |  |  |  |  |  |  |  |  | |  |
| Influenza Vaccine | 0.83 (0.55-1.26) | 0.38 | 0.54 (0.32-0.90) | 0.02 | 0.81 (0.3-2.16) | 0.67 | 0.4 (0.24-0.68) | 0.001 | 0.36 (0.19-0.68) | 0.002 | 0.27 (0.08-0.86) | | 0.03 |
| Gestational age at first prenatal visit | 1 (0.98-1.03) | 0.76 | 0.99 (0.95-1.04) | 0.75 | 1.05 (0.98-1.12) | 0.16 | 0.99 (0.92-1.06) | 0.77 | 0.94 (0.84-1.04) | 0.23 | 0.9 (0.76-1.05) | | 0.17 |
| Maternal age less than 19 years | 1.08 (0.69-1.69) | 0.73 | 1.09 (0.65-1.84) | 0.74 | 0.98 (0.45-2.15) | 0.96 | 1.25 (0.52-3.02) | 0.62 | 0.55 (0.24-1.23) | 0.14 | 0.3 (0.08-1.11) | | 0.07 |
| Maternal age more than 35 years | 1.19 (0.82-1.74) | 0.36 | 1.68 (1.02-2.75) | 0.04 | 2.07 (0.97-4.41) | 0.06 | 1.06 (0.51-2.2) | 0.88 | 0.95 (0.4-2.24) | 0.91 | 0.7 (0.19-2.55) | | 0.59 |
| Multiple births (current gestation) | 14.04 (8.42-23.4) | <0.001 | 14.48 (7.15-29.34) | <0.001 | 14.07 (4.81-41.15) | <0.001 | 11.21 (3.24-38.82) | <0.001 | 22.18 (4.31-114.15) | <0.001 | 74.05 (10.73-511.31) | | <0.001 |
| Maternal medical risk factors¶ | 1.23 (0.92-1.65) | 0.16 | 1.27 (0.87-1.85) | 0.21 | 1.88 (1.03-3.43) | 0.04 | 0.72 (0.44-1.16) | 0.17 | 0.74 (0.4-1.38) | 0.34 | 0.9 (0.38-2.14) | | 0.81 |
| Labor/delivery complications§ | 2.07 (1.61-2.68) | <0.001 | 2.1 (1.49-2.96) | <0.001 | 2.7 (1.57-4.64) | <0.001 | 1.66 (0.95-2.89) | 0.07 | 1.49 (0.75-2.97) | 0.26 | 1.59 (0.62-4.03) | | 0.33 |
| Birth defect | 2.29 (1.04-5.04) | 0.04 | 2.7 (1.03-7.05) | 0.04 | 1.73 (0.51-5.83) | 0.38 | 2.77 (0.75-10.28) | 0.13 | 1.77 (0.44-7.05) | 0.42 | 4.21 (0.58-30.5) | | 0.15 |
| Maternal diabetes | 1.46 (0.73-2.93) | 0.29 | 1.18 (0.51-2.71) | 0.69 | 0.52 (0.14-1.95) | 0.33 | 1.22 (0.36-4.14) | 0.75 | 1.34 (0.32-5.62) | 0.69 | 4.56 (0.4-52.62) | | 0.22 |
| Hypertension | 3.05 (1.87-4.98) | <0.001 | 3.03 (1.55-5.93) | 0.001 | 4.08 (1.48-11.25) | 0.007 | 2.18 (0.75-6.36) | 0.15 | 2.69 (0.74-9.85) | 0.13 | 3.66 (0.64-21.02) | | 0.15 |
| Mother insured | 1.2 (0.91-1.58) | 0.2 | 1.34 (0.92-1.95) | 0.13 | 1.44 (0.76-2.7) | 0.26 | 1.31 (0.71-2.42) | 0.38 | 1.79 (0.78-4.11) | 0.17 | 1.03 (0.34-3.14) | | 0.96 |
| Multivitamin use in pregnancy | 1.14 (0.87-1.5) | 0.35 | 1.16 (0.79-1.69) | 0.45 | 0.84 (0.46-1.54) | 0.57 | 1.3 (0.73-2.29) | 0.37 | 1.53 (0.74-3.16) | 0.25 | 0.7 (0.23-2.12) | | 0.53 |
| History of smoking during pregnancy | 1.31 (0.75-2.26) | 0.34 | 1.17 (0.61-2.27) | 0.64 | 0.66 (0.21-2.03) | 0.47 | 2.22 (0.82-5.97) | 0.12 | 4.21 (0.98-18.11) | 0.05 | 3.34 (0.64-17.39) | | 0.15 |
| History of alcohol use in pregnancy | 0.97 (0.41-2.28) | 0.94 | 1.22 (0.41-3.58) | 0.72 | 0.6 (0.07-5.1) | 0.64 | 0.8 (0.13-4.97) | 0.81 | 0.42 (0.04-3.9) | 0.44 | * | | |
| Black race | 1.44 (1.11-1.87) | 0.006 | 1.21 (0.84-1.75) | 0.3 | 1.05 (0.55-2.01) | 0.88 | 1.24 (0.69-2.24) | 0.47 | 0.96 (0.47-1.98) | 0.92 | 1.74 (0.67-4.49) | 0.25 | |
| Education less than 12th grade | 1.13 (0.73-1.74) | 0.59 | 1.24 (0.75-2.05) | 0.41 | 1.72 (0.67-4.41) | 0.26 | 0.87 (0.42-1.81) | 0.72 | 1 (0.42-2.35) | 1 | 2.36 (0.79-6.99) | 0.12 | |
| Mother married | 0.98 (0.73-1.31) | 0.89 | 0.94 (0.61-1.43) | 0.76 | 0.53 (0.27-1.02) | 0.06 | 0.98 (0.47-2.04) | 0.96 | 0.93 (0.35-2.47) | 0.89 | 2.39 (0.85-6.74) | 0.1 | |
| Maternal weight pre-pregnancy (kg) | 1 (1-1) | 0.74 | 1 (1-1.01) | 0.35 | 1.01 (1-1.01) | 0.02 | 1 (0.99-1.01) | 0.95 | 1 (0.99-1.01) | 0.87 | 0.99 (0.98-1.01) | 0.47 | |
| §Labor/delivery complications include abruptio placenta, anesthetic complications, breech presentation, cephalopelvic disproportion, cord prolapse, dysfunctional labor, excessive bleeding, febrile 100F/38C, fetal distress, moderate to heavy meconium staining, placenta previa, labor < 3-hours, PROM ,12-hours, labor >20-hours, seizures during labor | | | | | | | | | | | | | |
| ¶Medical risk factors include acute or chronic lung disease; anemia (hb<10 or hct<30); cardiac disease; diabetes; eclampsia; genital herpes; hemoglobinopathy; hydramnios/oligohydramnios; chronic hypertension; pregnancy induced hypertension; incompetent cervix; previous infant >4,000-g; previous preterm, small for gestational age, or low birth weight delivery; renal disease; Rh sensitization ; rubella; syphilis; uterine bleeding | | | | | | | | | | | | | |
| *Dropped from the model due to insufficient variability | | | | | | | | | | | | | |

| **Table S2: Complete results of secondary adjusted models for Small for Gestational Age** | | | | | | | | | | | | |
| --- | --- | --- | --- | --- | --- | --- | --- | --- | --- | --- | --- | --- |
| Covariate | All seasons/periods | | Putative influenza season (Oct-May) | | Pre-influenza activity period | | Period of at least local influenza activity | | Period of at least regional influenza activity | | Period of widespread influenza activity | |
|  | OR (95% CI) | p value | OR (95% CI) | p value | OR (95% CI) | p value | OR (95% CI) | p value | OR (95% CI) | p value | OR (95% CI) | p value |
|  |  |  |  |  |  |  |  |  |  |  |  |  |
| Influenza Vaccine | 0.96 (0.66-1.42) | 0.86 | 0.84 (0.5-1.4) | 0.5 | 1.02 (0.42-2.48) | 0.96 | 0.68 (0.32-1.46) | 0.33 | 0.7 (0.27-1.87) | 0.49 | 0.29 (0.09-0.91) | 0.04 |
| Gestational age at first prenatal visit | 1.04 (1-1.07) | 0.03 | 1.04 (0.99-1.08) | 0.09 | 1.07 (1-1.15) | 0.05 | 1.05 (0.99-1.11) | 0.08 | 1.01 (0.94-1.08) | 0.8 | 0.99 (0.88-1.12) | 0.87 |
| Maternal age less than 19 years | 1.09 (0.72-1.67) | 0.68 | 1.08 (0.61-1.91) | 0.79 | 1.31 (0.62-2.78) | 0.48 | 1.49 (0.7-3.16) | 0.3 | 1.65 (0.68-4.05) | 0.27 | 0.97 (0.25-3.73) | 0.96 |
| Maternal age more than 35 years | 0.82 (0.52-1.29) | 0.39 | 0.91 (0.5-1.68) | 0.77 | 0.43 (0.2-0.94) | 0.03 | 0.84 (0.28-2.52) | 0.75 | 0.42 (0.1-1.86) | 0.26 | 0.13 (0.03-0.5) | 0.004 |
| Multiple births (current gestation) | 5.3 (3.27-8.57) | <0.001 | 4.57 (2.47-8.44) | <0.001 | 4.71 (1.77-12.54) | 0.002 | 7.27 (1.89-28.02) | 0.004 | 1.7 (0.51-5.63) | 0.39 | 4.06 (0.73-22.46) | 0.11 |
| Maternal medical risk factors¶ | 1.09 (0.81-1.49) | 0.57 | 1.26 (0.82-1.94) | 0.28 | 1.41 (0.72-2.76) | 0.32 | 1.77 (0.9-3.48) | 0.1 | 1.56 (0.64-3.81) | 0.33 | 1.98 (0.51-7.65) | 0.32 |
| Labor/delivery complications§ | 1.07 (0.81-1.4) | 0.64 | 1.02 (0.7-1.48) | 0.92 | 0.83 (0.46-1.47) | 0.51 | 1.1 (0.61-1.98) | 0.74 | 1.59 (0.76-3.31) | 0.21 | 0.88 (0.25-3.14) | 0.85 |
| Birth defect | 0.83 (0.42-1.64) | 0.59 | 0.85 (0.33-2.19) | 0.73 | 0.63 (0.12-3.23) | 0.58 | 1.16 (0.19-7.16) | 0.87 | 0.97 (0.13-7.04) | 0.97 | 0.19 (0.01-3.51) | 0.27 |
| Maternal diabetes | 1.77 (0.68-4.6) | 0.24 | 1.68 (0.5-5.71) | 0.4 | 2.31 (0.37-14.41) | 0.37 | 1.76 (0.27-11.24) | 0.55 | 3.04 (0.3-30.59) | 0.34 | 17.62 (1.7-183.12) | 0.02 |
| Hypertension | 2.69 (1.57-4.62) | <0.001 | 3.02 (1.41-6.46) | <0.001 | 2.87 (0.92-8.92) | 0.07 | 4.33 (1.39-13.45) | 0.01 | 4.81 (1.18-19.64) | 0.03 | 1.65 (0.25-10.81) | 0.6 |
| Mother insured | 0.81 (0.61-1.08) | 0.16 | 0.71 (0.47-1.06) | 0.1 | 0.45 (0.24-0.86) | 0.02 | 1.1 (0.58-2.11) | 0.76 | 1.23 (0.63-2.4) | 0.55 | 0.91 (0.27-3.07) | 0.89 |
| Multivitamin use in pregnancy | 1.19 (0.89-1.59) | 0.24 | 1.03 (0.69-1.54) | 0.88 | 0.82 (0.42-1.6) | 0.56 | 1.06 (0.6-1.85) | 0.85 | 0.9 (0.48-1.67) | 0.73 | 1.28 (0.41-3.97) | 0.67 |
| History of smoking during pregnancy | 1.58 (0.95-2.62) | 0.08 | 1.92 (0.95-3.89) | 0.07 | 1.92 (0.68-5.46) | 0.22 | 0.68 (0.2-2.29) | 0.53 | 1.6 (0.41-6.25) | 0.5 | 0.9 (0.08-9.52) | 0.93 |
| History of alcohol use in pregnancy | 0.63 (0.26-1.54) | 0.31 | 0.86 (0.3-2.43) | 0.77 | 3.64 (0.78-17.07) | 0.1 | 0.24 (0.03-2.11) | 0.2 | * | | * | |
| Black race | 2.24 (1.69-2.97) | <0.001 | 2.48 (1.75-3.51) | <0.001 | 2.73 (1.62-4.6) | <0.001 | 3.3 (1.92-5.68) | <0.001 | 4.12 (2.1-8.07) | <0.001 | 4.76 (1.72-13.21) | 0.003 |
| Education less than 12th grade | 1.38 (0.94-2.03) | 0.1 | 1.62 (0.96-2.74) | 0.07 | 1.38 (0.7-2.72) | 0.36 | 1.21 (0.49-2.97) | 0.68 | 0.55 (0.21-1.45) | 0.23 | 0.3 (0.1-0.94) | 0.04 |
| Mother married | 0.99 (0.7-1.39) | 0.94 | 1.31 (0.84-2.05) | 0.24 | 1.29 (0.67-2.46) | 0.45 | 1.28 (0.65-2.53) | 0.47 | 1.2 (0.54-2.65) | 0.65 | 1.04 (0.36-3.05) | 0.94 |
| Maternal weight pre-pregnancy (kg) | 0.99 (0.99-0.99) | <0.001 | 0.99 (0.98-1) | 0.001 | 0.99 (0.99-1) | 0.02 | 0.99 (0.98-1) | 0.05 | 0.99 (0.98-1) | 0.16 | 1 (0.99-1.01) | 0.66 |
| §Labor/delivery complications include abruptio placenta, anesthetic complications, breech presentation, cephalopelvic disproportion, cord prolapse, dysfunctional labor, excessive bleeding, febrile 100F/38C, fetal distress, moderate to heavy meconium staining, placenta previa, labor < 3-hours, PROM ,12-hours, labor >20-hours, seizures during labor | | | | | | | | | | | | |
| ¶Medical risk factors include acute or chronic lung disease; anemia (hb<10 or hct<30); cardiac disease; diabetes; eclampsia; genital herpes; hemoglobinopathy; hydramnios/oligohydramnios; chronic hypertension; pregnancy induced hypertension; incompetent cervix; previous infant >4,000-g; previous preterm, small for gestational age, or low birth weight delivery; renal disease; Rh sensitization ; rubella; syphilis; uterine bleeding | | | | | | | | | | | | |
| *Dropped from the model due to insufficient variability | | | | | | | | | | | | |
